# Cardio-toxicity among patients with sarcoma: a cardio-oncology registry

**DOI:** 10.1186/s12885-020-07104-9

**Published:** 2020-06-30

**Authors:** Sivan Shamai, Zach Rozenbaum, Ofer Merimsky, Matthew Derakhshesh, Yonatan Moshkovits, Joshua Arnold, Yan Topilsky, Yaron Arbel, Michal Laufer-Perl

**Affiliations:** 1 Department of Oncology and Tel-Aviv Sourasky Medical Center, Tel Aviv University, Tel Aviv, Israel; 2 Sackler School of Medicine, Tel Aviv University, Tel Aviv, Israel; 3 Department of Cardiology, Tel-Aviv Sourasky Medical Center, Tel Aviv University, 64239 Tel Aviv, Israel

**Keywords:** Sarcoma, CTRCD, Cardiotoxicity, Echocardiography, GLS

## Abstract

**Background:**

Chemotherapy induced cardio-toxicity has been recognized as a serious side effect since the first introduction to anthracyclines (ANT). Cardio-toxicity among patients with breast cancer is well studied but the impact on patients with sarcoma is limited, even though they are exposed to higher ANT doses. The commonly used term for cardio-toxicity is cancer therapeutics related cardiac dysfunction (CTRCD), defined as a left ventricular ejection fraction (LVEF) reduction of > 10%, to a value below 53%. The aim of our study was to estimate the prevalence of CTRCD in patients diagnosed with sarcoma and to describe the baseline risk factors and echocardiography parameters among that population.

**Methods:**

Data were collected as part of the Israel Cardio-Oncology Registry (ICOR), enrolling all patients evaluated in the cardio-oncology clinic at our institution. The registry was approved by the local ethics committee and is registered in clinicaltrials.gov (Identifier: NCT02818517). All sarcoma patients were enrolled and divided into two groups - CTRCD group vs. non-CTRCD group.

**Results:**

Among 43 consecutive patients, 6 (14%) developed CTRCD. Baseline cardiac risk factors were more frequent among the non-CTRCD group. Elevated left ventricular end systolic diameter and reduced Global Longitudinal Strain were observed among the CTRCD group. During follow-up, 2 (33%) patients died in the CTRCD group vs. 3 (8.1%) patients in the non-CTRCD group.

**Conclusions:**

CTRCD is an important concern among patients with sarcoma, regardless of baseline risk factors. Echocardiography parameters may provide an early diagnosis of cardio-toxicity.

## Background

Soft tissue sarcomas (STS) are a relatively rare entity, with an estimated 13,040 new cases and 5150 deaths reported in the United States in 2018. Bone sarcomas (BS) are even less common, with an estimated incidence of 3450 cases and 1590 deaths a year [1]. Anthracyclines (ANT), mainly doxorubicin, is the cornerstone of systemic therapy in sarcoma. Protocols and dosage vary immensely in the different stages and subtypes of the disease. Nonetheless, patients with sarcoma are commonly exposed to high doses of ANT compared to other types of cancer [2]. Chemotherapy induced cardiac dysfunction has been recognized as a serious side effect since the first introduction to ANT in the 1960’s [3]. ANT is still considered to be the most significant cardio-toxic drug, as it is known to be dose-dependent [4] and irreversible [5]. According to the 2016 European Society of Cardiology position paper, the incidence of doxorubicin-related cardiac dysfunction was found to be 3–5% at a cumulative dose of 400 mg/m^2^ and 7–26% at a dose of 550 mg/m^2^ [4]. Though cardio-toxicity has been well studied among patients with breast cancer, [6] there is limited data regarding the risk for cardiac dysfunction and its related mortality among patients with sarcoma [7], even though the latter are exposed to higher doses of cardio-toxic chemotherapy. Echocardiography is the modality used most often to assess cardiac function among patients with cancer. According to the American and European Society of Echocardiography Expert Consensus, a left ventricular ejection fraction (LVEF) reduction of > 10%, to a value below 53% is defined as cancer therapeutics related cardiac dysfunction (CTRCD) [5]. Although LVEF is a sensitive marker for the development of cardiac dysfunction, previous literature has shown that significant LVEF reduction manifests only after substantial and mostly irreversible myocardial damage [8]. Therefore, novel techniques for early detection of myocardial damage are required.

The objectives of the current study were to estimate the prevalence of CTRCD in patients diagnosed with sarcoma and to describe the baseline risk factors and echocardiography parameters among that population.

## Methods

The study population is part of the Israel Cardio-Oncology Registry (ICOR) - a prospective registry enrolling all patients evaluated at the Tel Aviv Sourasky Medical cardio-oncology clinic. All patients signed an informed consent at the first visit and are then followed prospectively. The registry was approved by the local ethics committee (Identifier: 0228–16-TLV) and is registered in clinicaltrials.gov (Identifier: NCT02818517). As standard of care in our facility, all patients diagnosed with sarcoma who are planned for ANT therapy are referred for cardiac evaluation in the cardio-oncology clinic and are recommended to perform echocardiography at baseline, after cumulative dose of 240 mg/m2 and after every additional dose. With the completion of therapy patients are recommended to perform echocardiography follow-up every 3 month until one year since the last ANT dose. Certainly, additional echocardiography exams are performed in case of cardiac symptoms. The follow-up protocol may vary according to the cumulative dose.

From October 2016 to July 2018, 49 patients with sarcoma were evaluated, of which 6 patients were excluded due to lack of a second echocardiography. All study participants underwent a full medical history evaluation including chronic diseases and cardiac risk factors. In addition, all participants performed at least two echocardiography exams as described in the following section.

All patients were treated with Doxorubicin given by a 15 min infusion. The main protocols included Doxorubicin either alone or with Olaratumab, given at 75 mg/m2 on day 1 every 21 days, or Doxorubicin with Ifosfamide and Mesna (AIM regimen) which included 37.5 mg/m2/day of Doxorubicin and 3000 mg/m2/day of Ifosfamide, given in days 1 and 2 every 21 days. Dexrazoxane was given according to our practical use protocol; including patients treated with cumulative dose of Doxorubicin more than 300 mg/m^2^, or from lower doses in case of pre-existing reduced LVEF.

Patients were divided into 2 groups: The CTRCD group, which included all patients developing LVEF reduction of > 10%, to a value below 53% and the non-CTRCD group which included all the remaining patients.

All-cause mortality data were retrieved from the electronic records of the governmental population.

All trans-thoracic echocardiograms (TTE) were performed by the same vendor, technician and interpreting cardiologist using a General Electric (GE) system, model Vivid S70. Routine Left ventricle (LV) echocardiographic parameters included LV diameters, and LVEF [9]. Early trans-mitral flow velocity (E), late atrial contraction (A) velocity, deceleration time and early diastolic mitral annular velocity (septal and lateral e’) were measured in the apical 4-chamber view to provide an estimate of LV diastolic function [10]. The peak E/peak e’ ratio was calculated (septal, lateral and average) from the average of at least 3 cardiac cycles. Left Atrium (LA) volume index was calculated using the biplane area length method at end-systole [11]. Speckle-tracking echocardiography (STE) longitudinal evaluation was performed [12]. Before each acquisition, images were optimized for endocardial visualization by adjusting the gain, compress, and time-gain compensation controls. Images were acquired using high frame rate (> 50 frames/s) apical views (four, two, and three chambers) [13]. Images were stored digitally and used for offline analysis. Analysis was performed using STE software to measure global longitudinal strain (GLS) from images acquired using the above scheme and tracking within an approximately 5 mm wide region of interest, which is thinner than the default. LV boundaries were initialized in an end-systolic frame and then automatically tracked throughout the cardiac cycle. Manual corrections were performed to optimize boundary tracking. Normal peak GLS was defined as ≤ − 19% [14, 15] adhered to the standard benchmark set by previous studies.

All data were summarized and displayed as a mean (± standard deviation) for continuous variables and as a number (percentage) of patients for categorical variables. Continuous variables were tested for normal distribution using histograms and Q-Q Plots. All statistical analyses were performed with SPSS (IBM Corp. Released 2013. IBM SPSS Statistics for Windows, Version 22.0. Armonk, NY: IBM Corp).

## Results

**Fig. 1:**
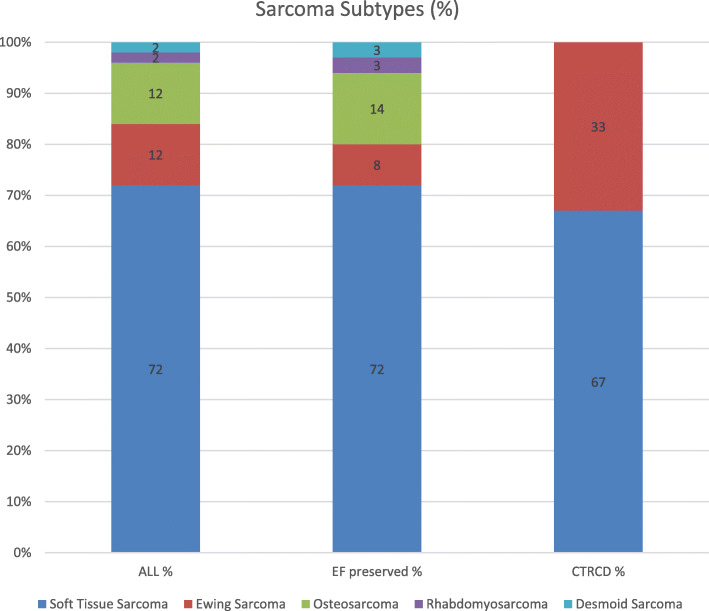
Sarcoma Subtypes according to cancer therapeutics related cardiac dysfunction (CTRCD)

Overall, 43 patients were included, with a female predominance (60.5%) and the mean age was 58(±16) years. The most common subtype of sarcoma was STS (72%) (Fig. 1) and 26 (60%) patients were metastatic (Table 1). The most common baseline risk factors were hypertension (37%) and hyperlipidemia (19%) and 11 (26%) patients were treated with baseline cardio-protective therapy including Angiotensin II receptor blockers (ARB), angiotensin-converting-enzyme inhibitors (ACEI) or beta blockers (BB). Only one patient had a history of ischemic heart disease (IHD) and the overall the mean LVEF (59 ± 2%) and GLS (− 20.3 ± 2.5%) were normal (Table 2).

**Table 1:** Cancer and chemotherapeutic parameters

	All (***n*** = 43)	non-CTRCD (***n*** = 37)	CTRCD (***n*** = 6)
**Soft Tissue Sarcoma** (n, %)	31 (72)	27 (72)	4 (67)
**Metastatic** (n, %)	26 (60)	21 (57)	5 (83)
**Anthracycline dose** mg/m^2^ (mean, SD)	337(±159)	328(±149)	388(±223)
**Dexrazoxane** (n, %)	25 (58)	21 (57)	4 (67)
**Ifosfamide** (n, %)	25 (58)	19 (51)	6 (100)

*CTRCD* cancer therapeutics related cardiac dysfunction, *SD* standard deviation

**Table 2:** Baseline characteristics according to cancer therapeutic related cardiac dysfunction (CTRCD)

	All (***n*** = 43)	non-CTRCD (***n*** = 37)	CTRCD (***n*** = 6)
**Age, years** (mean, SD)	58 ± 15	59 ± 14	48 ± 20
**Female Gender** (n, %)	26 (60.5%)	21 (57%)	5 (83%)
**Hypertension** (n, %)	16 (37%)	15 (41%)	1 (17%)
**Diabetes Mellitus** (n, %)	3 (7%)	3 (8%)	0 (0%)
**Hyperlipidemia** (n, %)	8 (19%)	8 (22%)	0 (0%)
**Past or Current Smoking** (n, %)	11 (26%)	10 (27%)	1 (17%)
**Ischemic Heart Disease** (n, %)	1 (2%)	1 (3%)	0 (0%)
**Atrial Fibrillation** (n, %)	2 (5%)	2 (5%)	0 (0%)
**Ischemic Stroke** (n, %)	2 (5%)	2 (5%)	0 (0%)
**Heart Rate, BPM** (mean, SD)	77.9(±13.6)	78.3(±14.1)	75(±9.8)
**Systolic Blood Pressure, mmHg** (mean, SD)	124.6(±17.7)	125.7(±17.3)	116.8(±20.2)
**Diastolic Blood Pressure, mmHg** (mean, SD)	70.5(±12.3)	70.9(±12.5)	67.6(±11.7)
**O2 Saturation,** % (mean, SD)	98.4(±1.9)	98.3(±2)	98.8(±1.3)
**Hemoglobin** g/dL (mean, SD)	11.3(±2.7)	11.5(±2.7)	10.3(±2.7)
**White Blood Cells** 10e3/μl (mean, SD)	7.9(±3.4)	7.7(±3.1)	9.4(±5.1)
**Red Blood Cell Distribution Width** % (mean, SD)	16.1(±2.4)	15.7(±2.3)	17.8(±2.6)
**Platelets** 10e3/μl (mean, SD)	256.5(±116.7)	233.9(±91.5)	381(±166.9)
**Creatinine** mg/dL (mean, SD)	0.8(±0.3)	0.8(±0.2)	0.6(±0.4)
**Beta Blockers** (n, %)	9 (21%)	6 (16%)	3 (50%)
**ACEI/ARBs** (n, %)	8 (18%)	7 (19%)	1 (17%)
**Statins** (n, %)	10 (23%)	10 (27%)	0 (0%)

*SD* standard deviation, *ACEI* angiotensin-converting-enzyme inhibitors, *ARB* Angiotensin II receptor blockers

Six patients (14%) were included in the CTRCD group, while the other 37 patients (86%) were included in the non-CTRCD group. Older age, male predominance and higher prevalence of baseline cardiac risk factors (including hypertension, diabetes mellitus, hyperlipidemia, smoking, atrial fibrillation and ischemic stroke) were present among the non-CTRD group (Table 2). None of the patients in the CTRCD group presented with multiple cardiovascular risk factors (≥2), comparing to 11(30%) patients in the non-CTRCD group. Regarding baseline chronic medications, BB therapy was higher among the CTRCD group, while statins therapy was among the non-CTCD group (Table 2).

Ewing sarcoma (Fig. 1) and metastatic disease (Table 1) were more frequent among the CTRCD group. Mean dose of cumulative ANT treatment was 337(±159) mg/m^2^ for all patients with 388(±223) mg/m^2^ for the CTRCD group compared to 328(±149) mg/m^2^ for the non-CTRCD group. Dexrazoxane and Ifosfamide therapy were higher among the CTRCD group. (Table 1).

All 43 patients performed two echocardiography exams with a mean interval of 131 days. Unfortunately, not all patients were compliant with the recommended exam at the recommended time and therefore at the time of the analysis only 29, 17, 13 and 9 patients performed 3rd, 4th, 5th and 6th echocardiography follows up. GLS was not the routine protocol in the beginning of the study and therefore only 22 of the patients performed GLS at first echocardiography evaluation. Higher left ventricle end systolic diameter (LVESD) (34 ± 4 mm vs. 28 ± 5 mm), a reduced GLS (-17.7 ± 2.1% vs. -20.7 ± 2.3%) and lower LV mass (134 ± 23 g vs. 170 ± 42 g) were noticed among the CTRCD group (Table 3). To confirm the accuracy of the LVESD difference we performed an inter-observer exam by evaluating the LVESD in 15 patients by a second independent observer and found that there was a high level of agreement between observers, with an interclass correlation coefficient (ICC) of 82% (*p* = 0.001). Diastolic function parameters (E/A, deceleration time, e’ lateral, e’ septal and E/e’ average) and right ventricular function (assessed by Tricuspid annular plane systolic excursion - TAPSE) were within the range of normal [10] in both groups (Table 3).

**Table 3:** Echocardiographic parameters according to cancer therapeutic related cardiac dysfunction (CTRCD)

	All (***n***-43)	non-CTRCD (***n*** = 37)	CTRCD (***n*** = 6)
**Ejection Fraction 1**^a^ % (mean, SD)	59(±2)	59(±2)	58(±4)
**Global Longitudinal Strain 1**^a^ % (mean, SD)	20.3(±2.5)	20.7(±2.3)	17.7(±2.1)
**Left Ventricular End Diastolic Dimension 1**^a^ mm (mean, SD)	48(±5)	48(±5)	50(±4)
**Left Ventricular End Systolic Dimension 1**^a^ mm (mean, SD)	29(±5)	28(±5)	34(±4)
**Left Ventricular mass 1**^a^ g (mean, SD)	166(±41)	170(±42)	134(±23)
**E/A 1**^a^ (mean, SD)	1.2(±0.6)	1.2(±0.7)	1.3(±0.6)
**Deceleration time 1**^a^ ms (mean, SD)	195(±46)	195(±48)	194(±30)
**e’ septal 1**^a^ cm/s (mean, SD)	7.4(±2.4)	7.2(±2.1)	9.6(±4.1)
**e’ lateral 1**^a^ cm/s (mean, SD)	9.7(±3.1)	9.3(±2.5)	13.1(±5.4)
**E/e’ septal 1**^a^ (mean, SD)	10.9(±3.4)	11.1(±3.4)	8.7(±2.2)
**E/e’ lateral 1**^a^ (mean, SD)	8.3(±2.5)	8.5(±2.4)	6.5(±2.4)
**E/e’ average 1**^a^ (mean, SD)	9.3(±2.6)	9.5(±2.6)	7.4(±2.3)
**Left Atrium Volume Index 1**^a^ mL/m2 (mean, SD)	34(±12)	34(±12)	36(±18)
**Tricuspid annular plane systolic excursion 1**^a^mm (mean, SD)	25(±4)	26(±4)	23(±3)
**Systolic Pulmonary Atrial Pressure, mmHg 1b**^a^ (mean, SD)	31(±15)	31(±16)	29(±4)

*SD* standard deviation

1^a^ are parameters measured at initial echocardiography evaluation

In the 3 months follow up after the diagnosis of CTRCD, 2 (33%) patients died in the CTRCD group vs. 3 (8.1%) patients in the non-CTRCD group. With respect to the causes of death, one patient died from heart failure, three patients died from non-cardiac causes, and one from unknown causes. Higher LVESD value was observed among the patients who deceased (33 ± 6 mm vs. 28 ± 5 mm). Among the six patients developing CTRCD, five (83%) were women and the mean age was 58(±16) years. Four patients were diagnosed with STS and two were diagnosed with Ewing Sarcoma, with 83% presenting with metastatic disease. Mean dose of cumulative ANT treatment was 388(±223) mg/m^2^ with 67% patients treated with Dexrazoxane. None of the patients had past therapy with ANT. Surprisingly, only one patient suffered from cardio-vascular risk factor (hypertension). The mean time for CTRCD diagnosis was 227 days (±114 days) from the beginning of chemotherapy, with 3 patients developing CTRCD after the completion of therapy and three patients during ANT therapy. All patients completed the chemotherapy as planned. A reduction in LVEF was observed from a mean of 58%(±4) to 47%(±4). Following the CTRCD development five patients started cardio-protective therapy (three patients BB, one ACEI and one BB + ACEI). Only one patient did not receive any cardio-protective therapy due to hypotension. Follow up echocardiography showed that in three patients the LVEF remained the same, in two patients an additional LVEF reduction was observed and in one patient the LVEF normalized without cardio-protective therapy. Two patients developed congestive heart failure, which led to mortality in one patient. Another patient died from a non-cardiac cause.

## Discussion

We performed a prospective evaluation of cardio-toxicity development among patients with sarcoma treated with ANT therapy. We observed a 14% incidence of CTRCD, which is higher comparing to previous studies reporting an incidence of less than 5% [4].

When trying to characterize the CTRCD group comparing to the non-CTRCD we observed a higher female predominance and surprisingly a younger age (Table 2). Previous studies [4, 5] suggested that a comprehensive pre-treatment evaluation may unveil predisposing cardiac risk factors and identify patients at risk for CTRCD development. Surprisingly, in our study, higher prevalence of baseline cardiac risk factors and multiple cardio-vascular risk factors were more common among the non-CTRCD group. This raises the question of whether routine cardiac assessment of all patients with sarcoma treated with ANT is advised, since there were no specific baseline risk factors to warrant CTRCD development. There are also questions as to the efficacy of pre-treatment cardio protective therapies. In the PRADA [16] and OVERCOME [17] trials, routine baseline use of ARB, BB and ACEI provided protection against early decline in LV function. Interestingly, in our study, baseline treatment with ARB or ACEI did not protect patients from CTRCD development. Furthermore, BB therapy was higher among the CTRCD group. This discrepancy can be explained through the understanding that the treatment in our study was not given for the purposes of preventing LV dysfunction, but rather as a treatment for chronic diseases (hypertension, ischemic heart disease, diabetes, etc.), which place those patients at a higher risk for LV dysfunction development. Moreover, once CTRCD was diagnosed treatment with ACEI and/or BB did not improve the LVEF based on follow-up echocardiography, thereby corroborating the mechanism of irreversible damage [5]. Interestingly, higher prevalence of statin therapy was observed among the non-CTRCD group. Past studies [18] have implied that statin therapy is associated with lower risk for incident heart failure among breast cancer patients treated with ANT; however randomized larger studies are needed.

Since the toxicity of ANT is considered to be irreversible [3], a routine echocardiographic evaluation during the treatment-course possibly enables the early diagnosis of cardiac dysfunction. In our study, bassline higher LVESD, lower LV mass and reduced GLS were observed among the CTRCD group. Past studies similarly imply that ANT exposure is associated with a decline in LV mass [19], as well as reduced GLS [20]. To our knowledge, there is no data regarding the predictive value of enlarged LVESD for CTRCD development among patients with cancer. Regarding reduced GLS, only 22 of the patients performed GLS at first echocardiography evaluation. Therefore, we believe that this number is too small and studies with a larger sample size are needed.

The CTRCD group presented with a more advanced disease and higher dose of mean ANT with a range from 100 mg/m2 and up to 450 mg/m2. There was no significant difference between the groups regarding the use of Dexrazoxane. This raises the question whether dexrazoxane should be introduced earlier in the protocol for CTRCD prevention, especially as past concerns regarding its toxicity are now abated [21]. All six patients with CTRCD were treated with Ifosfamide as well. While heart failure has been described as a side effect of Ifosfamide, it is less common than ANT induced cardio-toxicity. A recent European phase III trial compared Ifosfamide based regimens to Cisplatin and Docetaxel in 693 lung cancer patients. Cardio-toxicity was diagnosed less frequently among patients treated with Ifosfamide (3% vs. 6.4%), usually with grade 1–2 [22].

The strengths of this study are its prospective nature, the focus given to a rare and under-studied patients population and the unity we achieved by performing the echocardiography exams by the same vendor, technician and interpreting cardiologist in order to prevent inter-vendor variability.

Our study has several limitations. First, it was a single center, observational study. Second, we acknowledge that the relatively small number of outcomes reduces the statistical power of our results and therefore we focused on describing the characteristics of the groups without performing univariable and multivariable logistic regression. Furthermore, considering the multiple numbers of analyses performed in this limited cohort, without the adjusting for multiple comparisons; requires that all results should be cautiously interpreted. However, since sarcoma is a rare type of cancer our study is considered to be relatively large. Finally, the relative short period of follow-up might have influenced the results, with the possibility of LVEF reduction and mortality occurring later in the course of follow up.

## Conclusions

In summary, CTRCD is frequent among patients diagnosed with sarcoma and treated with ANT, regardless of baseline risk factors. There is a need for early diagnosis of cardiotoxicity in order to prevent its development. In our study we evaluated echocardiography parameters that may be associated with CTRCD development that might be used to identify early cardio-toxicity injury.

## Data Availability

Not provided due to local institute ethics committee’s restrictions regarding external distribution.

## Abbreviations

STS: Soft tissue sarcomas
BS: Bone sarcomas
ANT: Anthracycline
LVEF: Left ventricular ejection fraction
CTRCD: Cancer therapeutics related cardiac dysfunction
ICOR: Israel Cardio-Oncology Registry
TTE: Trans-thoracic echocardiography
GE: General Electric
LV: Left ventricle
LA: Left Atrium
STE: Speckle-tracking echocardiography
GLS: Global longitudinal strain
ARB: Angiotensin II receptor blocker
ACEI: Angiotensin converting enzyme inhibitor
BB: Beta blocker
LVESD: Left ventricle end systolic diameter
LVEDD: Left ventricle end diastolic diameter
TAPSE: Tricuspid annular plane systolic excursion
ICC: Interclass correlation coefficient
